# Mortality and treatment response amongst HIV-infected patients 50 years and older accessing antiretroviral services in South Africa

**DOI:** 10.1186/s12879-018-3083-z

**Published:** 2018-04-10

**Authors:** Halima Dawood, Razia Hassan-Moosa, Nonhlanhla-Yende Zuma, Kogieleum Naidoo

**Affiliations:** 1 Centre for the AIDS Programme of Research in South Africa (CAPRISA), Durban, South Africa; 2 Infectious Diseases, Department of Internal Medicine, Greys Hospital, Pietermaritzburg, South Africa; 3 MRC-CAPRISA HIV-TB Pathogenesis and Treatment Research Unit, Doris Duke Medical Research Institute, University of KwaZulu-Natal, Durban, South Africa

**Keywords:** HIV, Older, Anti-retroviral treatment, Mortality, South Africa

## Abstract

**Background:**

Little is known about the clinical presentation and outcomes amongst older HIV infected populations accessing ART in sub-Saharan Africa. We compared mortality amongst HIV infected patients accessing ART that were <  50 years to those ≥50 years in Kwa-Zulu Natal, South Africa.

**Methods:**

We undertook a retrospective review of medical records of patients that accessed HIV services at the CAPRISA AIDS Treatment program (CAT) between June 2004 to December 2012 (*N* = 4003). HIV infected patients, 14 years or older were enrolled. All-cause mortality and treatment response to ART in those < 50 years to those ≥50 years were compared. A Kaplan-Meier curve and log-rank test were used to compare the cumulative probability of death between the two age groups with the primary endpoint being mortality. Statistical analysis was done using SAS (version 9.4.; SAS Institute Inc., Cary, NC, USA).

**Results:**

Of 4003 individuals, 262 (6.5%) were ≥ 50 years (older group). The median age in those ≥50 years and <  50 year was 54.5 and 32.0 years, respectively. The younger group was mainly female (64.7%). There was no difference in mortality rate, between the older (6.9/100 person-years (py), 95% confidence interval (CI): 4.7–9.6) and younger group (5.3/100 py, 95% CI: 4.7–5.8) at 60 months (*p* = 0.137). In the multivariable model older patients had a significantly higher risk of death compared to younger patients. (hazard ratio (HR) 1.60, 95% CI: 1.08–2.39, *p* = 0.019).The rate of CD4+ cell count increase was higher in those < 50 years (β = 0.34, 95% CI: 0.19–0.50, *p* < 0.001) with no difference in viral suppression. The older group showed significantly higher prevalence of diabetes (6.3%) and hypertension (21.5%), *p* < 0.001.

**Conclusion:**

ART initiation in older HIV infected patients was associated with a higher mortality compared to those younger than 50 years. ART immunological response was less robust in older individuals. The increase in hypertension and diabetes among older patients suggests the need to restructure and integrate primary and specialized health care services into ART services.

**Electronic supplementary material:**

The online version of this article (10.1186/s12879-018-3083-z) contains supplementary material, which is available to authorized users.

## Background

In 2015, there were approximately 36.7 million people living with human immunodeficiency virus (HIV) [1]. North American and European HIV treatment cohorts have demonstrated good clinical outcomes among older individuals initiating antiretroviral treatment (ART) [2, 3]. However, an increase in long-term treatment adverse events and non-HIV related disease has been reported [4]. The ATHENA (AIDS Therapy Evaluation in The Netherlands) cohort in Netherlands comprising 10,000 patients on ART with a median age of 44 years, has postulated that the proportion of HIV-infected patients 50 years and older will increase from 28% in 2010 to 73% in 2030, [5] by which time the median age of patients will increase to 56.6 years [5].

South Africa had approximately 6.4 million HIV infected people in 2012 accounting for 17% of the global burden of HIV infection [6]. It is anticipated that the successes in HIV treatment and prevention over the past decade in South Africa and Sub-Saharan Africa attributable to the rapid expansion of ART access, stabilizing HIV sero-prevalence and increase in life expectancy from 52 years in 2003 to 61 years 2011, will create a demographic shift of those in HIV care [7].

Specifically an increased number of HIV infected patients surviving to older age groups. Indeed, the number of adults over the age of 60 years in Africa is projected to rise by 55% between 2010 and 2025, a 135% increase since 1995 [8, 9]. It is estimated that by 2050, approximately 15% of the total South African population will be 60 years or older [10]. Recent South African HIV sero-prevalence studies among patients ≥50 years report HIV prevalence rates of 7.6% (95% CI 6.5–8.8) in 2012 [6].

Current ART use has contributed to a dramatic reduction in opportunistic infection incidence [11]. However, as the median age of those on ART increases, the clinical course of HIV disease is likely to differ in older individuals compared to younger patients. While, better ART adherence and good virologic response has been demonstrated in those ≥50 years, studies have also showed significantly slower immunologic recovery among older patients [12, 13]. In the United States older HIV infected individuals are at an increased risk for co-morbidities associated with life-style, aging and complications of long term ART use such as hypertension, diabetes, and renal impairment [14]. Treatment of HIV in older patients in Sub-Saharan Africa is poorly understood largely as limited resources are currently directed at scaling up services for pregnant women, children, and young adults [15]. The HIV infected aging population in Sub Saharan Africa will continue to grow, thus there is a critical need to understand HIV and ART related co- morbidities and outcomes in these patients to enable resources for early identification and prompt and appropriate management.

We conducted a retrospective data review of patients enrolled and followed up prospectively on ART at the Center for AIDS Programme of Research in South Africa (CAPRISA) AIDS treatment (CAT) programme between 2004 and 2012 and aimed to compare survival and treatment outcomes of patients < 50 years to those ≥50 years in Kwa-Zulu Natal, South Africa.

## Methods

### Study design and participants

#### Study patients, design and setting

The CAPRISA Acquired immunodeficiency syndrome (AIDS) treatment program (CAT) was a prospective treatment cohort, which enrolled patients between June 2004 to December 2012 (*N* = 4006) at two treatment sites, the CAPRISA eThekwini clinical research site (urban) adjacent to the Prince Cyril Zulu Communicable Disease Centre (PCZCDC), in Durban, and the Vulindlela site, (rural), in KwaZulu-Natal. Details of the cohort, procedures and the primary outcomes of the cohort have been described previously [16]. ART-naïve patients, 14 years and older, who initiated standard first-line ART (two nucleoside reverse transcriptase inhibitors and a non-nucleoside reverse transcriptase inhibitor) between June 2004 and December 2012 as per current national guidelines [17, 18], were included in this analysis. In 2004 the eligibility to initiate ART was a CD4+ count of less than 200 cells/mm^3^ or an AIDS defining illness. The CD4+ count threshold was increased to ≤350 cells/mm^3^ for pregnant women and any CD4+ count level for those with TB co-infection in 2010. Finally, in 2012 the CD4+ count threshold was increased to ≤350 cells/mm^3^. Inadditon, all pregnant women irrespective of CD4 + cell count level were eligible for ART initiation in 2012 [17].

HIV-infection was confirmed by two successive rapid HIV Enzyme linked immunoabsorbent assay (ELISA) tests. Loss to follow-up was defined as missing three or more consecutive scheduled visits and where attempts to locate the patient in the community failed. Death was ascertained through telephonic or physical tracking of patients and confirmed by family members.

### Study procedures

ART eligibility and treatment guidelines followed the existing national guidelines that prevailed [17]. Patient information including demographic information, detailed medical history and a full evaluation of the current clinical condition was undertaken at screening, ART enrolment and initially monthly for the first 6 months post-ART initiation, then every 2–3 months, unless clinically indicated. Routine safety laboratory tests and CD4+ counts (FACS flow cytometer: Becton Dickinson, Franklin Lakes NJ, USA) and viral loads (Roche Cobas Amplicor HIV-1 Monitor v1.5) were done at baseline and six-monthly. These details, along with details of intercurrent illnesses and medication prescribed, were captured in real-time on case report form (CRF) templates and submitted to a central CAPRISA electronic data management system (Clinical DataFax Systems Inc., Ontario, Canada). Queries arising during validation of the data were recorded in quality control reports sent to the sites on a regular basis. All patients received standard of care as per national department of health guidelines [17].

Virologic failure was defined as a viral load > 1000 copies/ml detected on two occasions, taken at least 4 weeks apart, and resulted in complete regimen change. Viral suppression or undetectable viral load was defined as a viral load of < 400 copies/ml. Those with a viral load below 1000 but ≥400 copies/ml were not considered as virological failure requiring complete ART regimen change.

Hypertension was defined as a blood pressure (BP) of 140 mmHg or more systolic or a diastolic BP of 90 mmHg or more at the time of ART initiation. Patients already on medication for hypertension were considered to be hypertensive. Patients were classified as diabetic if they had a fasting plasma glucose of 7 mmol/l (126 mg/dL) or random plasma glucose > 11.1 mmol/l (200 mg/dL) or if on insulin or any oral hypoglycemic agent at the time of ART initiation.

#### Ethics and consent

Ethics approval for use of the treatment program data was obtained from the Biomedical Research Ethics Committee of the University of KwaZulu-Natal.(Ref-E248/05). The biomedical research ethics committee reviewed the application and deemed the need for consent unnecessary in terms of national regulations: “*the REC may approve a waiver of consent for secondary use of material or data where no more than minimal risk of harm is likely; and donor rights and welfare interests are unlikely to adversely affected; and research cannot be conducted if waiver is not approved”.*Informed consent was obtained for HIV testing and treatment (including those less than 16 years) from all individuals included in the study in keeping with standard of care [18]. The national health Act permitted children 12 years and older to access HIV care without parental or guardian consent. Hence no parental or guardian consent was required for this age group [18].

### Statistical analysis

This analysis included all patients 14 years and older who were followed-up for a maximum of 60 months post ART initiation. Analysis was stratified by age at ART initiation, < 50 years [younger] and ≥ 50 years [older]. Demographic and clinical variables were summarized using median with interquartile range (IQR), mean with standard deviation and percentages. All statistical tests were two sided. Fisher’s exact test was used for the analysis of categorical data. The t-test for independent samples or Wilcoxon two-sample test was used for the analysis of continuous data.

Linear mixed model was used to determine the difference in rate of increase in CD4+ cell count between the two age groups. Square root transformation was used to achieve CD4+ cell count normality.

A Kaplan-Meier curve and log-rank test were used to compare the cumulative probability of death between the two age groups with the primary endpoint being mortality. Censorship was at the earliest recorded date of death, date of loss to follow-up or date of transfer out of the cohort. Poisson approximations were used to calculate confidence intervals (CIs) for mortality rates. Univariate and multivariate proportional hazards regression models were used to assess predictors of mortality. Multivariable models were adjusted for the following baseline covariates: gender, clinic site, CD4+ cell count, body mass index (BMI), World Health Organisation (WHO) stage, diabetes status, hypertension status, past history of tuberculosis (TB) and current tuberculosis disease. Proportionality was assessed by fitting time dependent covariates in a model created by interacting baseline variables with survival time. All-cause mortality was assessed. Statistical analysis was done using SAS (version 9.4.; SAS Institute Inc., Cary, NC, USA).

## Results

### Patient characteristics

A total of 4003 individuals initiated antiretroviral treatment (ART) between June 2004 and December 2012; 6.5% were 50 years and older (older group). The baseline demographic characteristics of the two groups are illustrated in Table 1. The median age at ART initiation in the older group was 54.5 years (interquartile range (IQR): 52–58 years) in comparison to 32.0 years (IQR: 28–38 years) in the younger group. The median duration of follow up for the cohort was 16.2 months (IQR: 8.0–34.9 months). The follow up of participants over time is reflected in Additional file 1: Figure S1.

**Table 1:** Baseline and demographic characteristics of individuals (*n* = 4003) older and younger with HIV infection on antiretroviral treatment (ART) from June 2004 to December 2012

Characteristics	Older (≥ 50 years) *N* = 262	Younger (< 50 years) *N* = 3741	*p*-value
Year of entry			–
2004	2 (0.8)	74 (2.0)	
2005	34 (13.0)	568 (15.2)	
2006	39 (14.9)	574 (15.3)	
2007	18 (6.9)	270 (7.2)	
2008	11 (4.2)	181 (4.8)	
2009	34 (13.0)	369 (9.9)	
2010	60 (22.9)	877 (23.4)	
2011	54 (20.6)	655 (17.5)	
2012	10 (3.8)	173 (4.6)	
Age (years), median (IQR)	54. 5 (52–58)	32 (28–38)	–
Gender, n (%)			
Male	117 (44.7)	1321 (35.3)	0.003
Female	145 (55.3)	2420 (64.7)	
Clinic site, n (%)			0.021
Urban	99 (37.8)	1691 (45.2)	
Rural	163 (62.2)	2050 (54.8)	
Median body mass index (kg/m^2^), (IQR)^a^	23.1 (20.5–27.1)	22.8 (20.2–26.4)	0.154
Baseline CD4+ count, (cells/μl) median(IQR)^b^	142 (86–197)	124 (60–184)	< 0.001
CD4+ cell count, cells/mm^3^: n (%)			0.119
< 200	173 (76.5)	2747 (81.2)	
200–350	45 (19.9)	568 (16.8)	
> 350	8 (3.5)	70 (2.1)	
Viral load (log copies/ml), mean (SD)^e^	4.9 (1.0)	5.0 (0.9)	0.268
WHO stage, n (%)^c^			0.623
1	40 (15.3)	556 (14.9)	
2	60 (22.9)	744 (20.0)	
3	131 (50.0)	2004 (53.8)	
4	31 (11.8)	424 (11.4)	
Past history of TB, n (%)^d^	62 (24.2)	1077 (29.5)	0.075
Prevalent TB, n (%)	62 (23.7)	907 (24.2)	0.882
Co-morbidities			
Hypertension^e^	55 (21.5)	79 (2.2)	<.001
Diabetes^f^	16 (6.3)	24 (0.7)	<.001
Occupation^g^			0.014
Employed	83 (32.2)	1193 (32.3)	
Unemployed	156 (60.5)	2358 (63.9)	
Scholar/Other	19 (7.4)	137 (3.7)	
Initial ART regimen			
2NRTI + NNRTI	260 (100%)	3718 (99.6%)	
2NRTI + PI	0	15 (0.4%)	

^a^221 missing BMI (8 older, 213 younger), ^b^392 missing CD4 count(36 older, 356 younger), ^c^13 younger missing WHO stage, ^d^102 missing past history of TB (6 older,96 younger), ^e^475 missing viral load (34 older,441 younger), ^e^96 missing hypertension (6 older,90 younger), ^f^96 missing diabetes (6 older,90 younger), ^g^57 missing occupation(4 older, 53 older)

There was no significant difference between the groups overall in terms of body mass index, baseline viral load, WHO clinical stage or current or past history of TB. Gender distribution between the groups varied, with the younger group comprising 64.7% female compared to 55.3% in the older group, *p* = 0.003.There were 28 pregnant women in this cohort. More older individuals (62.2%) was found in the rural site compared to 54.8%, in the urban site, (*p* = 0.021). Most patients in both groups presented with WHO clinical stage 3 or 4 disease prior to initiating ART. A significantly higher prevalence of diabetes (6.3%) and hypertension (21.5%) was observed in the older group (*p* < 0.001), Table 1.

Overall, approximately 73% of patients presented with a baseline CD4+ cell count of < 200 cells/mm^3^, with older patients demonstrating a significantly higher median baseline CD4+ cell count compared to younger patients,142 vs.124 cells/mm^3^, *p* = 0.001 (Table 1). The changes in baseline CD4+ cell count thresholds at different years of ART initiation is illustrated in Additional file 1: Figure S2.

### Response to ART

**Fig. 1:**
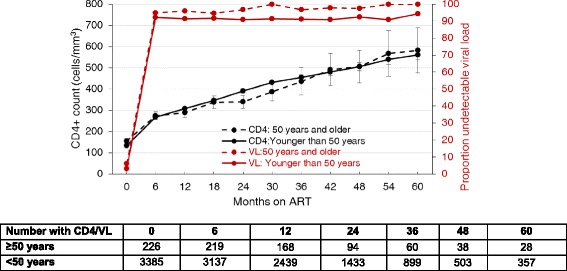
Mean CD4+ count over with 95% CI and proportion with undetectable viral load

The rate of CD4+ cell count increase was significantly higher in patients < 50 years old (β = 0.345, 95% CI 0.189–0.4500, *p* < 0.001) compared to those > 50 years, Fig. 1, Additional file 2: Table S3. This trend persisted after adjusting for baseline covariates (β = 0.34 95% CI 0.18–0.50, *p* < 0 .001). The proportion of individuals that achieved virologic suppression during follow-up did not differ between the groups, (Fig. 1). At the end of follow- up there were 15/357(4.2%) individuals in the younger group and none in the older group with a CD4 + cell count < 200 cells/μl. None of the older patients experienced virological failure whilst 0.4% of the younger group required a change of ART treatment regimen due to first regimen virologic failure.

### Mortality

**Fig. 2:**
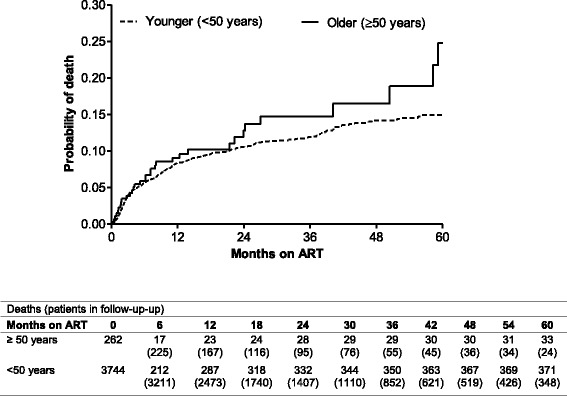
Kaplan-Meier estimates of cumulative probability of death for patients exposed to ART according to age groups

There were a total of 403 deaths in the study at 60 months, with 8.2% of all deaths belonging to patients in the older group and 91.8% among patients in the younger group. The mortality rate was 6.7 per 100 person-years (py) (95% confidence interval (CI): 4.6–9.4) in the older group and 5.1 per 100 person-years (95% CI: 4.6–5.7) (*p* = 0.137) (Fig. 2) in the younger group. Most patients died within the first 24 months of follow-up (Fig. 2). The probability of death was similar at 6,12 and 24 months between the groups, however, between 24 months and 60 months the probability of death was higher in the older group, 6.7 vs 5.1 per 100 person years, respectively, p = 0.137 (Fig. 2, Additional file 1: Figure S3). While the cause of death in the majority of cases, was unknown, where a cause of death was established, the proportion of non HIV/TB associated deaths was higher in those 50 years and older. (Additional file 2: Table S1).Two-thirds of the known causes of death in those less than 50 years were associated with HIV with or without TB, while only 6% of deaths in those 50 years and older were reported to be HIV/TB associated.

Age group was not associated with mortality in the univariate model. In the multivariable model older patients had a significantly higher risk of death compared to younger patients. (hazard ratio (HR) 1.60, 95% CI: 1.08–2.39, *p* = 0.019). An increase in CD4+ cell count and body mass index was associated with a decline in the risk of mortality.

Other variables associated with a higher risk of mortality included low BMI, and WHO stage 3 and 4 disease, a diagnosis of prevalent diabetes and the absence of prevalent TB at ART initiation. WHO clinical stage 4 was associated with a fourfold risk of mortality. These factors together with male gender and hypertension were significant in the sensitivity analysis (Table 3) that assumed that all patients lost to follow up had died (Table 2).

**Table 2:** Patient outcomes at 60 months post ART initiation

	Older (≥ 50 years) *N* = 262	Younger (< 50 years) *N* = 3741	*p*-value
Died	33 (12.6)	370 (9.9)	0.167
Transferred out	203 (77.5)	2850 (76.2)	0.653
Relocated	2 (0.8)	51 (1.4)	0.580
Defaulted/stopped ART	23 (8.8)	458 (12.2)	0.115
Other^a^	1 (0.4)	12 (0.3)	0.586

^a^3 were in jail and 8 reasons unknown

## Discussion

In this prospective, observational cohort comparing outcomes of HIV infected individuals among patients 50 years and older to those younger than 50 years, we observed a higher mortality in the older compared to those younger than 50 years following 60 months of treatment. Unsurprisingly, most deaths occurred in the first 24 months post ART initiation. The mortality in the first 12 months did not differ significantly in both groups and is in keeping with other reports from sub Saharan Africa where high mortality is reported following initial initiation of ART [19].

Cause of death, where established was found to be not related to HIV or TB in the vast majority of patients older than 50 years of age in our study. Conversely, almost two-thirds of all deaths were found to be associated to either TB and/or HIV among patients that were less than 50 years old. These findings illustrate the benefit of ART in protecting against AIDS related morbidity and mortality over time. Furthermore, older patients had an overall higher prevalence of co-morbidity such as hypertension and diabetes at ART initiation [20].

We found that irrespective of age, men were more likely to die within the ART programme compared to women. This however may, in part, be reflecting the epidemiology of HIV in Sub-Saharan Africa. Here women acquire HIV at a younger age and enroll in ART programs earlier in the course of their disease through enhanced contact with health care services that traditionally offer an entry point into the HIV care continuum such as sexual reproductive health services, antenatal and perinatal services [21]. This difference in mortality outcomes by gender has been demonstrated previously in a rural South African setting, however the data presented was limited to pre-ART and 1 year post ART initiation outcomes only [7]. Similarly, prevalent TB also previously shown to be associated with a low mortality, maybe related to TB care being an access point to earlier ART initiation.

Our study also demonstrated that patients ≥50 years demonstrated poorer immununologic recovery on ART, as shown by poorer CD4+ cell count recovery, compared to younger patients. The slow CD4+ cell count recovery in our older cohort is consistent with other studies which indicate that older individuals have a lower rate of increase in CD4+ cell counts following ART exposure compared to younger individuals [22]. This ability to increase CD4 cell count determined the risk of death in the multivariable model [23, 24]. The risk of death was associated with CD4+_+_ cell count. Without CD4+ cell count in the model the difference in risk of death between the older and younger group was insignificant and hence CD4 + cell count at ART initiation is an important predictor of mortality in those ≥50 years.

Traditional age related co-morbidities appear to occur earlier in older HIV infected patients compared to HIV uninfected counterparts of the same age [14]. We saw a ten-fold increased prevalence of hypertension and diabetes in the older compared to younger cohort. Co - morbidities such as hypertension and diabetes were more prevalent in the older group and this may reflect an increased likelihood of contact with health care services and thus earlier initiation of ART in accordance with HIV treatment guidelines. However, the risk of co-morbidities increases as individuals get older, and there is a need to modify antiretroviral treatment to ensure a minimum impact on these comorbidities [14].

At baseline we noted a nine fold increase in prevalent diabetes in older patients in comparison to the younger cohort and a similar trend in hypertension prevalence.(Table 3). A recent study in KwaZulu Natal estimated the crude prevalence of diabetes to be 12.5% and this is higher than the national prevalence of 9,2% [25]. This high prevalence is reflected in this HIV infected cohort.

**Table 3:** Predictors of all-cause mortality

Variable at ART initiation	Original analyses				Sensitivity analyses^a^			
	Univariate		Multivariate		Univariate		Multivariate	
	HR (95% CI)	*p*-value	HR (95% CI)	*p*-value	HR (95% CI)	*p*-value	HR (95% CI)	*p*-value
Group								
< 50 years	1.0		1.0		1.0		1.0	
≥ 50 years	1.29 (0.91–1.85)	0.157	1.60 (1.08–2.39)	0.019	0.98 (0.75–1.29)	0.908	1.07 (0.79–1.44)	0.652
Gender								
Male	1.0		1.0		1.0		1.0	
Female	1.42 (1.16–1.73)	<.001	1.14 (0.91–1.44)	0.242	1.41 (1.23–1.61)	<.001	1.31 (1.12–1.53)	<.001
Site								
Urban	1.0		1.0		1.0		1.0	
Rural	1.24 (1.01–1.53)	0.043	1.20 (0.94–1.53)	0.147	1.53(1.32–1.78)	<.001	1.49 (1.25–1.78)	<.001
CD4+ count (per 50 cells/mm^3^ increase)	0.73 (0.67–0.78)	<.001	0.78 (0.72–0.84)	<.001	0.90 (0.86–0.94)	<.001	0.93 (0.89–0.98)	0.003
Body mass index (per 5 kg/m^2^ increase)	0.66 (0.58–0.74)	<.001	0.76 (0.66–0.87)	<.001	0.77(0.71–0.83)	<.001	0.84 (0.77–0.91)	<.001
WHO stage								
Stage 1	1.0		1.0		1.0		1.0	
Stage 2	1.50 (0.94–2.40)	0.091	1.49 (0.89–2.48)	0.129	0.83 (0.65–1.06)	0.14	0.84 (0.64–1.10)	0.212
Stage 3	2.47(1.65–3.71)	<.001	2.12 (1.34–3.36)	0.001	1.15 (0.94–1.40)	0.181	1.18 (0.93–1.49)	0.172
Stage 4	5.01 (3.23–7.75)	<.001	4.18 (2.51–6.95)	<.001	1.62 (1.26–2.07)	<.001	1.72 (1.28–2.32)	<.001
Presented with diabetes								
No	1.0		1.0		1.0		1.0	
Yes	1.55 (0.69–3.48)	0.286	2.67 (1.16–6.14)	0.021	1.08 (0.56–2.08)	0.821	1.83 (0.94–3.60)	0.077
Presented with hypertension								
No	1.0		1.0		1.0		1.0	
Yes	0.50 (0.24–1.05)	0.067	0.57 (0.26–1.24)	0.158	0.51 (0.31–0.84)	0.008	0.58 (0.34–1.00)	0.049
Past history of TB								
Yes	1.23 (1.00–1.52)	0.051	0.89 (0.70–1.12)	0.323	1.03 (0.89–1.19)	0.732	0.91 (0.78–1.08)	0.291
No	1.0		1.0		1.0		1.0	
Prevalent TB								
Yes	1.0		1.0		1.0		1.0	
No	1.24 (0.98–1.64)	0.068	1.82 (1.34–2.45)	<.001	1.23 (1.03–1.46)	0.020	1.41 (1.15–1.75)	0.001

^a^Assuming all patients who were loss to follow-up died

Whilst the prevalence of hypertension and diabetes appear to be lower than the general population, this cohort illustrates the opportunity that HIV treatment provides for comprehensive multi-morbidity care in Sub Saharan Africa.A similar prevalence was found in a Malawi cohort [26]. Presenting for HIV care enables screening and early detection and treatment for hypertension and diabetes.

The strategies of anti-retroviral therapy (SMART) and evaluation of subcutaneous Proleukein ® in randomized International trial (ESPIRIT) trials demonstrated that the frequency of Non-AIDS events was approximately 50% higher than AIDS events and the mortality of non AIDS events were double that of AIDS events [27]. In this cohort the prevalence of TB was similar to hypertension at baseline in the older cohort compared to the younger cohort. The prevalence of hypertension and diabetes was much lower than the prevalence of TB (> 10 times) in the younger cohort indicating that the focus of screening in the different age groups may be different over the life course of treatment of HIV infection [28].

### Limitations

This study has the advantage of a large sample size; however, survival bias may have resulted in patients with a better prognosis entering the cohort especially in the older group. Furthermore, the proportion of those older than 50 years was smaller than those in the younger group. This disproportion may have further contributed to bias. Furthermore, the change in ART eligibility guidelines and CD4+ cell counts (Additional file 1: Figure S2) over the follow up time likely contributed to a time dependent bias. Missing data as listed in Table 1 may have contributed to bias but the sensitivity analysis (Table 3) did not show any significant changes in the predictors of mortality. Whilst the enrollment into the treatment cohort was between 2004 to 2012, participants exited to facilities nearer their homes during follow up, hence not all participants were followed up for the entire study duration. The median time for transfer out of the cohort was 2 years (IQR:1 to 3 years). Furthermore, missing data for parameters of renal function, causes of death and ART adverse events limits a more in-depth analysis of co-morbidities and its association with survival and ART outcomes in the older cohort [29].

## Conclusion

We demonstrate that following ART initiation, older HIV infected Africans had a higher mortality compared to those younger than 50 years. Response to ART treatment in terms of virological response is similar to younger individuals on ART; however the immunological response is less robust in older individuals. This cohort highlights the need for multi-morbidity management and the benefits of ART over time.. Further studies are required to determine the causes of mortality in those older than 50 years and the long term effect of ART on co-morbidities and pharmacokinetics of ART in those 50 years and older to optimize long term safety. In addition, the impact of convergence of non-communicable diseases, HIV infection, chronic ART and ageing in this population in the long term needs to be evaluated. The increase in medical co-morbidities will require co-ordination and restructuring of primary and specialized care services in this vulnerable population and may require a different integrated model of care.

## Additional files

Additional file 1: **Figure S1.** Flow diagram of cumulative participant attrition over 6 years following enrolment into the CAPRISA Acquired immunodeficiency syndrome (AIDS) treatment program (CAT) with HIV infection on antiretroviral treatment (ART) from June 2004 to December 2012. **Figure S2.** CD4+ count at ART initiation at different years of enrolment reflecting time dependent bias of increasing CD4+ count thresholds at ART initiation. **Figure S3.** Kaplan-Meier estimates of cumulative probability of death during the first 6 months for patients exposed to ART according to age groups (log rank *p* = 0.90). (DOCX 236 kb)

Additional file 2: **Table S1.** Causes of death in both groups. **Table S2.** Mortality rate over time by age. **Table S3.** Mean CD4+ count over time and mean increase in relation ART initiation measurements. (DOCX 27 kb)

## Abbreviations

AIDS: Acquired immunodeficiency syndrome
ART: Antiretroviral treatment
ATHENA: AIDS Therapy Evaluation in The Netherlands
BMI: Body mass index
BP: Blood pressure
CAT: CAPRISA AIDS Treatment program
CD4: Cluster Differentiation
CI: Confidence interval
CRF: Case report form
DHHS: Department of Health and Human Services
ELISA: Enzyme linked immunoabsorbent assay
ESPIRIT: Evaluation of subcutaneous Proleukein ® in randomized International trial
HIV: Human immunodeficiency virus
HR: Hazard ratio
IQR: Interquartile range
PCZCDC: Prince Cyril Zulu Communicable Disease Centre
Py: Person-years
SMART: Strategies of anti-retroviral therapy
START: Strategic Timing of AntiRetroviral Treatment
TB: Tuberculosis
TEMPRANO: Early Antiretroviral Treatment and/or Early Isoniazid Prophylaxis Against Tuberculosis in HIV-infected Adults
WHO: World Health Organisation
