# Supplementary material for: Mortality and treatment response amongst HIV-infected patients 50 years and older accessing antiretroviral services in South Africa
Source: BMC Infect Dis. 2018 Apr 10;18:168. doi: 10.1186/s12879-018-3083-z (PMC5894176; doi:10.1186/s12879-018-3083-z)
Supplement: Supplementary file 1 — Figure S1. Flow diagram of cumulative participant attrition over 6 years following enrolment into the CAPRISA Acquired immunodeficiency syndrome (AIDS) treatment program (CAT) with HIV infection on antiretroviral treatment (ART) from June 2004 to December 2012. Figure S2. CD4+ count at ART initiation at different years of enrolment reflecting time dependent bias of increasing CD4+ count thresholds at ART initiation. Figure S3. Kaplan-Meier estimates of cumulative probability of death during the first 6 months for patients exposed to ART according to age groups (log rank p = 0.90). (DOCX 236 kb) [file 12879_2018_3083_MOESM1_ESM.docx]

**Additional file 1: Figures**

**Excluded (N=45)**

<14 years (n=3)

Age or date of birth not recorded (n=42)

Started ART between June 2004 and December 2012

(N=4048)

Included in the analyses (N=4003)

**Did not reach 1 year (N=1366)**

310 Died

254 Defaulted

25 Relocated

772 Transferred out

5 Other

Reached 1 year

(N=2637)

**Did not reach 2 years (N=2502)**

359 Died

367 Defaulted

39 Relocated

1726 Transferred out

11 Other

Reached 2 years

(N=1501)

**Did not reach 3 years (N=3097)**

378 Died

410 Defaulted

43 Relocated

2255 Transferred out

11 Other

Reached 3 years

(N=906)

**Did not reach 4 years (N=3448)**

396 Died

446 Defaulted

49 Relocated

2546 Transferred out

11 Other

Reached 4 years

(N=555)

**Did not reach 5 years (N=3631)**

403 Died

469 Defaulted

52 Relocated

2696 Transferred out

11 Other

Reached 5 years

(N=372)

Reached 6 years

(N=180)

**Additional file 1: FigureS1 Flow diagram of cumulative participant attrition over six years following enrolment into the CAPRISA Acquired immunodeficiency syndrome (AIDS) treatment program (CAT) with HIV infection on antiretroviral treatment (ART) from June 2004 to December 2012.**

**Additional file 1: Figure S2 CD4+ count at ART initiation at different years of enrolment reflecting time dependent bias of increasing CD4+ count thresholds at ART initiation.**

**Additional file 1: Figure S3 Kaplan-Meier estimates of cumulative probability of death during the first six months for patients exposed to ART according to age groups. (log rank p =0.90)**
