# Supplementary material for: Mortality and treatment response amongst HIV-infected patients 50 years and older accessing antiretroviral services in South Africa
Source: BMC Infect Dis. 2018 Apr 10;18:168. doi: 10.1186/s12879-018-3083-z (PMC5894176; doi:10.1186/s12879-018-3083-z)
Supplement: Supplementary file 2 — Table S1. Causes of death in both groups. Table S2. Mortality rate over time by age. Table S3. Mean CD4+ count over time and mean increase in relation ART initiation measurements. (DOCX 27 kb) [file 12879_2018_3083_MOESM2_ESM.docx]

Supplementary tables

**Additional file 2: Table S1:** Causes of death in both groups

| 50 years and older | | Younger than 50 years |
| --- | --- | --- |
| HIV | 1 (3.0%) | 62 (16.7%) |
| HIV/TB | 1 (3.0%) | 16 (4.3%) |
| TB | 2 (6.1%) | 51 (13.7%) |
| Non HIV/TB | 7 (21.2%) | 45 (12.1%) |
| Unknown | 22 (66.7%) | 197 (53.1%) |
| Total | **33** | **371** |

**Additional file 2: Table S2**: Mortality rate over time by age

|  | **Older**  **(≥ 50 years)** | | | **Younger**  **(< 50 years)** | | |  | |
| --- | --- | --- | --- | --- | --- | --- | --- | --- |
| **Follow-up** | **No of deaths** | **Person-years** | **Mortality rate (95% CI)** | **No of deaths** | **Person-years** | **Mortality rate (95% CI)** | **Rate ratio (95% CI)** | **p-value** |
| Month 6 | 15 | 120.95 | 12.4  (6.9 -20.5) | 208 | 1737.88 | 12.0 (10.4 -13.7) | 1.04  (0.62 -1.76) | 0.883 |
| Month 12 | 22 | 217.44 | 10.1  (6.3 -15.3) | 285 | 3126.90 | 9.1 (8.1 -10.2) | 1.11  (0.72 -1.71) | 0.637 |
| Month 18 | 24 | 285.59 | 8.4 (5.4 -12.5) | 313 | 4152.43 | 7.5 (6.7 -8.4) | 1.11  (0.73 -1.68) | 0.622 |
| Month 24 | 27 | 338.91 | 8.0 (5.3 -11.6) | 330 | 4950.54 | 6.7 (6.0 -7.4) | 1.20  (0.81 -1.78) | 0.362 |
| Month 30 | 29 | 381.69 | 7.6 (5.1 -10.9) | 342 | 5586.94 | 6.1 (5.5 -6.8) | 1.24  (0.85 -1.81) | 0.266 |
| Month 36 | 29 | 414.13 | 7.0 (4.7 -10.1) | 348 | 6092.58 | 5.7 (5.1 -6.3) | 1.23  (0.84 -1.8) | 0.284 |
| Month 42 | 30 | 438.97 | 6.8 (4.6 -9.8) | 362 | 6468.69 | 5.6 (5.0 -6.2) | 1.22  (0.84 -1.77) | 0.295 |
| Month 48 | 30 | 459.32 | 6.5 (4.4 -9.3) | 366 | 6756.46 | 5.4 (4.9 -6.0) | 1.21  (0.83 -1.76) | 0.316 |
| Month 54 | 31 | 476.53 | 6.5 (4.4 -9.2) | 368 | 6997.97 | 5.3 (4.7 -5.8) | 1.24  (0.86 -1.79) | 0.250 |
| Month 60 | 33 | 491.21 | 6.7 (4.6 -9.4) | 370 | 7194.26 | 5.1 (4.6 -5.7) | 1.31  (0.92 -1.87) | 0.137 |

**Additional file 2: Table S3:** Mean CD4+ count over time and mean increase in relation ART initiation measurements

|  | **Mean CD4+ count (95%CI)** | | **Mean CD4+ change from ART initiation**  **(95% CI)** | |
| --- | --- | --- | --- | --- |
| **Months post ART initiation** | **Older (≥50 years)** | **Younger (< 50 years)** | **Older (≥50 years)** | **Younger (< 50 years)** |
| 0 | 154.2 (140.4-167.9) | 132.9 (129.0-136.8) |  |  |
| 6 | 274.4 (256.3-292.6)  (N=219) | 268.0 (262.7-273.2)  (N=3137) | 109.9 (92.9 -127.0) | 126.8 (122.1 -131.6) |
| 12 | 289.5 (266.4-312.6)  (N=168) | 307.4 (300.9-313.8)  (N=2439) | 141.3 (119.7 -163.0) | 178.7 (172.6 -184.8) |
| 24 | 339.3 (307.8-370.7)  (N=94) | 390.7 (381.3-400.2)  (N=1433) | 204.3 (173.4 -235.2) | 274.8 (265.5 -284.0) |
| 36 | 436.3 (371.9-500.8)  (N=60) | 453.9 (440.5-467.4)  (N=899) | 308.0 (239.9 -376.1) | 340.6 (326.9 -354.2) |
| 48 | 506.3 (429.8-582.9)  (N=38) | 505.4 (485.9-524.8)  (N=503) | 373.6 (290.7 -456.5) | 397.0 (377.3 -416.8) |
| 60 | 581.9 (475.5-688.3)  (N=28) | 560.7 (537.0-584.4)  (N=357) | 448.4 (322.9 -574.0) | 452.1 (427.9 -476.3) |

**_A two month window period on either side of each annual visit was used for a record of CD4+ cell count or viral load. In addition CD4+ cell counts were done biannually but annual estimates are reported._**
